# Endoscopic mucosal resection with an over‐the‐scope clip for colorectal tumors (with video)

**DOI:** 10.1002/deo2.70076

**Published:** 2025-03-17

**Authors:** Takahiro Muramatsu, Tomoaki Tashima, Tomonori Kawasaki, Tsubasa Ishikawa, Kodai Esaki, Kei Sugimoto, Masami Sano, Shotaro Ishizaka, Yumi Mashimo, Takao Itoi, Shomei Ryozawa

**Affiliations:** ^1^ Department of Gastroenterology Saitama Medical University International Medical Center Saitama Japan; ^2^ Department of Gastroenterology Tokyo Medical University Hospital Tokyo Japan; ^3^ Department of Pathology Saitama Medical University International Medical Center Saitama Japan

**Keywords:** colorectal tumors, endoscopic full‐thickness resection, endoscopic mucosal resection, endoscopic submucosal dissection, over‐the‐scope clip

## Abstract

**Background:**

Endoscopic mucosal resection (EMR) and endoscopic submucosal dissection may result in complications or may be unsuitable for tumors that are difficult to treat endoscopically. We investigated the usefulness of a newly developed endoscopic resection technique—EMR with an over‐the‐scope clip (EMR‐O)—for difficult‐to‐treat lesions.

**Method:**

We retrospectively examined patients who underwent EMR‐O for colorectal tumors between September 2017 and January 2024. Patient and lesion characteristics, technical success rates, en bloc resection rates, R0 resection rates, procedure time, histopathology, and the clinical course were evaluated.

**Results:**

EMR‐O was performed for 18 patients. Indications for EMR‐O included residual or recurrent lesions (seven patients; 38.9%), diverticulum lesions (five patients; 27.8%), appendiceal orifice lesions (three patients; 16.7%), T1 cancers (two patients; 11.1%), and subepithelial tumors (one patient; 5.5%). The median lesion size was 11 mm. The rates of technical success, en bloc resection, and R0 resection were 100%, 86.7%, and 86.7%. The median procedure time was 10 min. The only adverse event was diverticulitis (one patient; 5.5%). Intraoperative and delayed perforation and bleeding were not observed. The pathological resection depths were full‐thickness for three patients (16.7%), muscularis resection for four patients (22.2%), and deep submucosal resection for 11 patients (61.1%).

**Conclusion:**

Although EMR‐O is limited by the target lesion size, it shortens the procedure time, prevents perforation, and avoids the need for surgery. EMR‐O may be a minimally invasive treatment option for small lesions that are difficult to treat endoscopically.

## INTRODUCTION

Endoscopic mucosal resection (EMR) and endoscopic submucosal dissection (ESD)^1^ are standardized techniques that use an endoscope to resect colorectal tumors. The en bloc resection rates are 89%–97% for ESD and 34.9%–53.2% for EMR.^2^, ^3^, ^4^, ^5^, ^6^ ESD has improved resection strategies,^7^, ^8^, ^9^, ^10^ thus making it possible to approach large lesions and lesions in difficult‐to‐resect locations. Furthermore, with the advent of underwater EMR (UEMR),^11^ it is now possible to treat lesions approximately 20 mm in size, which are difficult to treat using conventional EMR/ESD.^12^, ^13^ However, even with these techniques, intradiverticular and appendiceal orifice lesions have a high risk of perforation, and residual or recurrent lesions after endoscopic treatment are difficult to treat endoscopically because of severe fibrosis, which causes their lack of elevation.^14^, ^15^, ^16^, ^17^, ^18^ Recently, endoscopic full‐thickness resection (EFTR) via a full‐thickness resection device (FTRD; Ovesco Endoscopy, Tubingen, Germany) has been used in Europe and the United States for difficult‐to‐treat endoscopic lesions.^19^, ^20^ The FTRD comprises a cap with an over‐the‐scope clip (OTSC; Ovesco Endoscopy)^21^ that is pre‐attached to a snare. The technical success rate using the FTRD is reportedly 88.2–89.5%.^19^, ^20^, ^22^, ^23^, ^24^ Because the FTRD enables a one‐step procedure, and because the lesion and surrounding environment cannot be confirmed during resection, the certainty of the procedure is questionable; consequently, it has not been approved in Japan. Therefore, we developed a novel endoscopic resection method—EMR using the OTSC (EMR‐O)—that is based on the same concept as that of FTRD^25^ but allows for the presence of the entire lesion and surrounding environment to be confirmed and resected using two steps. We previously reported the usefulness of EMR‐O for duodenal neuroendocrine tumors (NETs),^26^ subepithelial tumors (SETs),^27^ and residual tumors after endoscopic resection.^28^ EMR‐O has a high en bloc resection rate, short operative time, and no major adverse events. The colon is associated with several lesions that are difficult to treat, such as colonic diverticula and appendiceal orifice; because EMR‐O can preserve the bowel, we considered it a particularly useful minimally invasive treatment method in this anatomical area. This study aimed to examine the usefulness and safety of EMR‐O for colorectal tumors that are difficult to treat endoscopically.

## PATIENTS AND METHODS

### Study design

This single‐center retrospective study was conducted at the Saitama Medical University International Medical Center. This study complied with the ethical guidelines of the Declaration of Helsinki and was approved by the Ethics Review Committee of Saitama Medical University International Medical Center (application number: 19–315).

Written informed consent was obtained from all patients after the risks and benefits of the treatment had been explained prior to performing EMR‐O.

### Participants

Between September 2017 and January 2024, 18 consecutive patients with colorectal lesions who underwent EMR‐O at our institute were enrolled in this study. The indications for EMR‐O were as follows: (1) recurrent or residual lesions after endoscopic resection; (2) locations with a high risk of perforation (appendiceal orifice, colonic diverticula); (3) SETs located within the submucosal layer observed using endoscopic ultrasonography; and (4) preoperative diagnosis of suspected deep submucosal invasive cancer in patients who are not considered suitable to undergo surgery. EMR‐O was performed for lesions ≤15 mm because they were considered suctioned within the cap of the OTSC. For lesions > 15 mm but ≤25 mm, hybrid EMR‐O or ESD was performed because the entirety of these lesions cannot be suctioned within the cap of the OTSC. Hybrid EMR‐O was defined as a planned piecemeal resection technique using EMR‐O and other EMR procedures. Hybrid EMR‐O was performed instead of ESD for lesions involving diverticula because of the possibility of perforation. For lesions > 25 mm, ESD or surgery was performed. Because of the risk of appendicitis, lesions involving the appendiceal orifice were limited to post‐appendectomy cases. All EMR‐O procedures were performed as inpatient procedures by three endoscopists (Takahiro Muramatsu, Tomoaki Tashima, and Tsubasa Ishikawa). At the time of this study, Tomoaki Tashima had performed > 200 OTSC procedures. Because Takahiro Muramatsu and Tomoaki Tashima had performed fewer than five OTSC procedures at the time of this study, Tomoaki Tashima supervised their EMR‐O procedures.

To examine the usefulness of EMR‐O, we retrospectively investigated the patient background, tumor characteristics, treatment results, complications, and clinical course.

### Procedure

#### Pre‐treatment simulation

Assuming that the delivery and placement of an OTSC was possible, when EMR‐O was considered, a transparent hood was attached to the tip of the scope during pretreatment using detailed endoscopy to imitate an OTSC, confirm insertion and operability at the lesion site, determine whether the lesion could be aspirated in the transparent hood, and perform a preliminary simulation of EMR‐O.

#### Treatment preparation and setting

The procedure was performed under intravenous anesthesia using 2–5 mg midazolam and 35 mg pethidine hydrochloride. We used the t‐type OTSC. The clip width was 11/6t if the scope was used for the upper gastrointestinal tract; however, it was 12/6t or 14/6t if the scope was used for the lower gastrointestinal tract. Local injections were not administered. We used a 10‐mm‐diameter snare (Captivator II; Boston Scientific, Marlborough, USA) with the 11/6t OTSC; however, we used a 15‐mm‐diameter snare (Snare Master; Olympus) with the 12/6t and 14/6t OTSCs. Regarding the output settings of the high‐frequency device (VIO3; Erbe, Tubingen, Germany), marking was performed using Soft coag (Effect 3, 40 W), and resection was performed using Endo Cut Q (Effect 3, Duration 1, and Interval 3).

#### Description of EMR‐O

**FIGURE 1:**
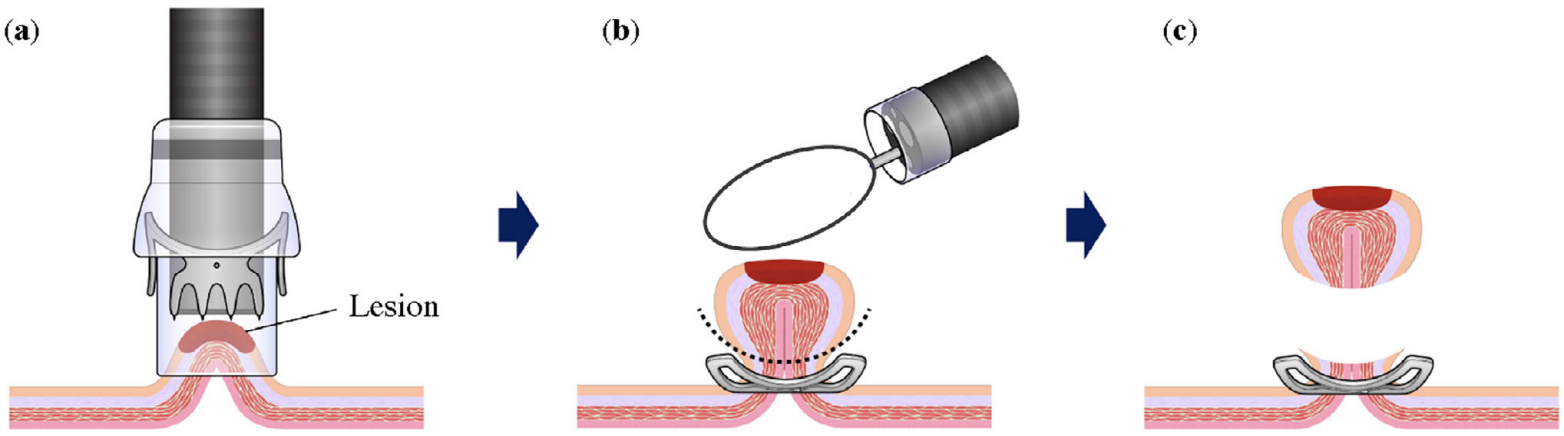
Schema of endoscopic mucosal resection (EMR) with an over‐the‐scope clip (OTSC; EMR‐O). (a) Aspiration of the lesion in the cap of the OTSC and deployment of the clip. (b) Snaring is performed just above the clip while the margin of the lesion is confirmed. (c) Resection of the lesion.

**FIGURE 2:**
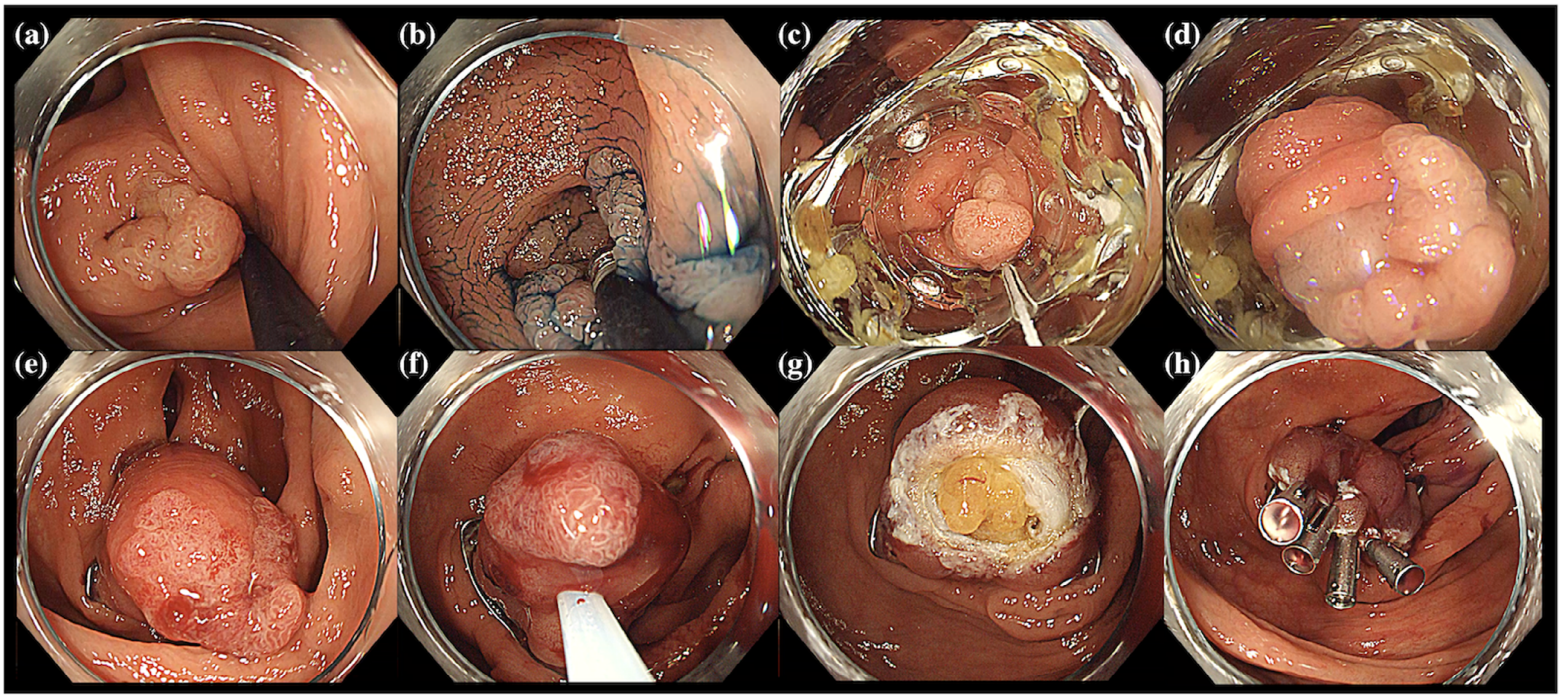
Endoscopic mucosal resection (EMR) with an over‐the‐scope clip (OTSC; EMR‐O) for the diverticulum lesion. (a) White light image. Lower gastrointestinal endoscopy shows a protuberant lesion extending into and out of the diverticulum in the ascending colon (diameter, 15 mm). (b) Indigo carmine dye. image. The flat lesion extends inside the diverticulum. (c) The OTSC is delivered to the lesion. (d) Aspiration of the lesion in the cap of the OTSC. (e) After releasing the clip, the diverticulum is inverted and the entire lesion is visible. (f) Snaring is performed just above the clip. (g) After lesion resection, the extraintestinal fatty tissue is observed on the resection surface. (h) The resected surface is closed using clips.

EMR‐O is a simple procedure comprising two steps: in step 1, an OTSC is placed under the lesion (Figure 1a), and the gastrointestinal wall is sutured and elevated in all layers (Figure 1b); and in step 2, the entire lesion is snared and resected just above the OTSC while the margin of the lesion is confirmed (Figure 1c). The actual procedure is shown in Figure 2 and Video S1.

#### Description of hybrid EMR‐O

**FIGURE 3:**
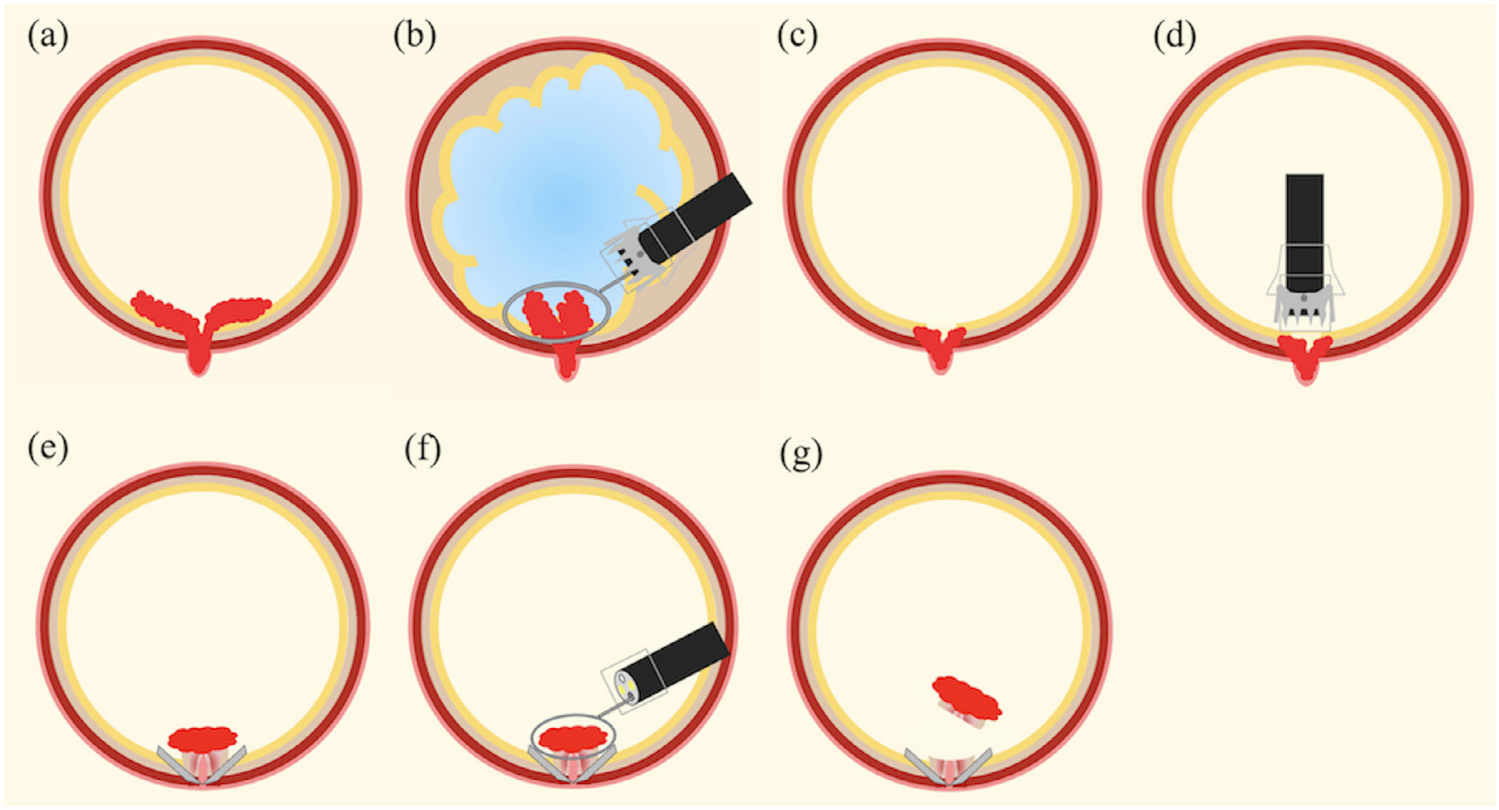
Schema of hybrid endoscopic mucosal resection (EMR) with an over‐the‐scope clip (OTSC; EMR‐O). (a) The lesion extends into and out of the diverticulum. (b) Underwater EMR is performed for the lesion outside the diverticulum. (c) The remaining lesion within the diverticulum. (d) The OTSC is delivered to the lesion. (e) The remaining lesion is aspirated in the applicator cap, and the OTSC is placed. (f) EMR‐O is performed. (g) Complete resection of the lesion.

**FIGURE 4:**
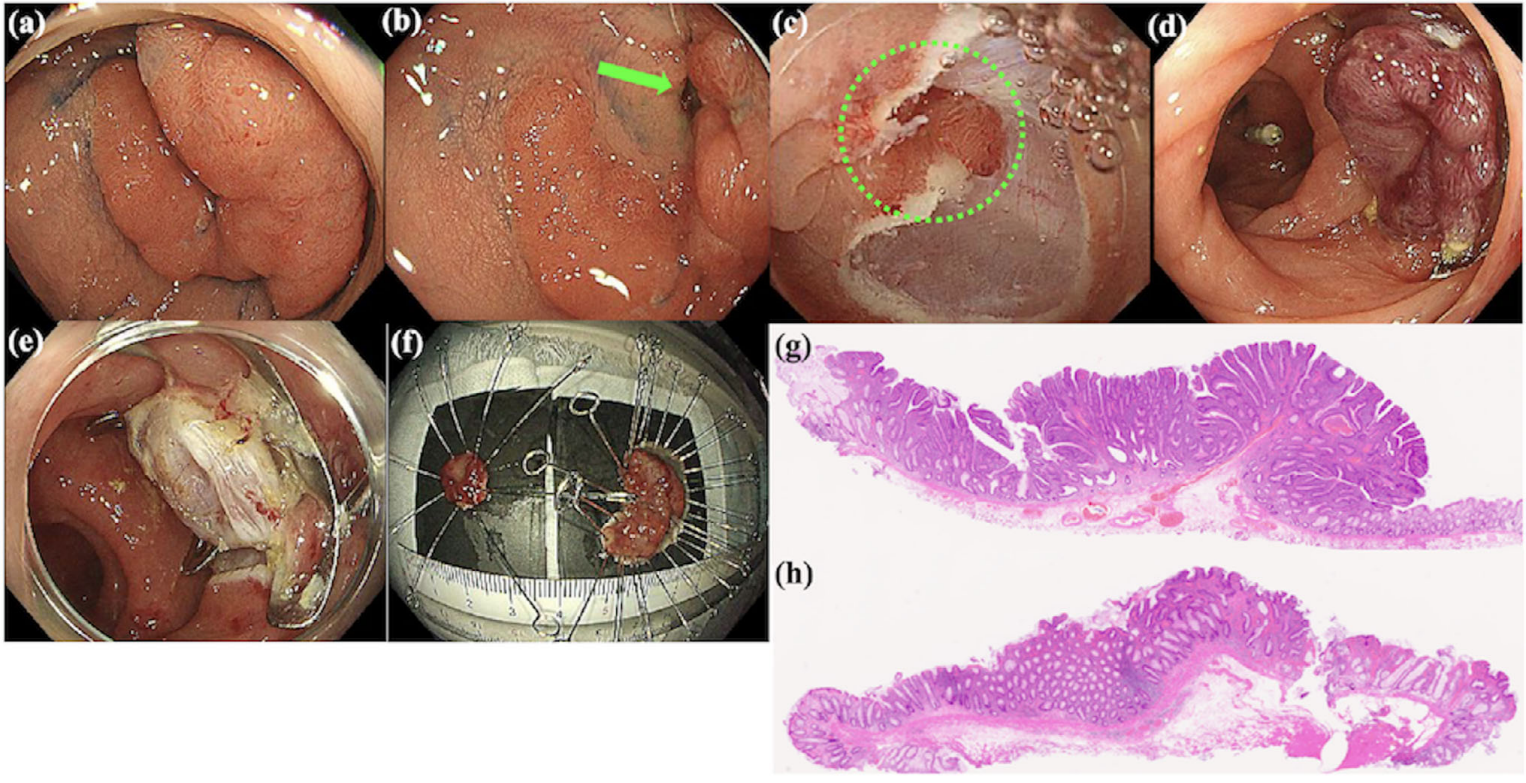
Hybrid endoscopic mucosal resection (EMR) with an over‐the‐scope clip (OTSC; EMR‐O) for the lesion that spread inside and outside the diverticulum. (a) A bifurcated lesion is located on the sigmoid colon (diameter, 25 mm) (b) A diverticulum in the center of the branch (green arrow). (c) After underwater EMR (UEMR) of the lesion outside the diverticulum, the lesion within the diverticulum remains (green dotted line). (d) The lesion within the diverticulum is aspirated in the cap of the OTSC, and the clip is released. (e) The lesion above the clip is resected. (f) Macroscopic image of the specimen. (g) Histopathological image of the specimen resected using UEMR (outside the diverticulum). The tumor was diagnosed as an intramucosal adenocarcinoma with no lymphovascular invasion. The horizontal and vertical margins were deemed negative. (h) Histopathological image of the specimen resected using EMR‐O (inside the diverticulum). The tumor was diagnosed as adenoma.

Hybrid EMR‐O is intended for lesions that can be systematically resected as two sections using EMR‐O and other EMR techniques (Figure 3). For cases involving lesions that spread both inside and outside the diverticulum (Figure 4a,b), lesions outside the diverticulum were resected using UEMR (Figure 4c), and lesions inside the diverticulum were resected using EMR‐O (Figure 4d,e).

#### Study outcomes

The main outcomes of this study were technical success, en bloc resection, and R0 resection rates. Technical success was defined as successful lesion resection with EMR‐O. En‐bloc resection was defined as macroscopic resection of the entire tumor as one piece. R0 resection was defined as the histopathological absence of tumor remnants in the horizontal and vertical margins of the resected specimen. The margins were categorized as HM0/VM0 (no tumor remnant in the horizontal/vertical margins), HM1/VM1 (residual tumor in the horizontal/vertical margins), and HMX/VMX (difficulty evaluating the horizontal/vertical margins). The procedure time, which was defined as the time from when the OTSC reached the target lesion to the time when the lesion was resected, and adverse events (such as delayed bleeding, which was defined as hematochezia requiring endoscopic hemostasis) were secondary outcomes. Furthermore, we histopathologically evaluated the resection depth. Deep submucosal resection was defined as the vertical margin of the resected specimen containing most of the submucosal layer. Muscular resection was defined as the vertical margin of the resected specimen containing the muscle layer. Full‐thickness resection was defined as the vertical margin of the resected specimen containing the serosa.

Quantitative data were expressed as the median (interquartile range) or number (percentage). All analyses were conducted using SPSS version 24.0 (SPSS Inc.).

## RESULTS

### Patient and lesion characteristics and indications for EMR‐O

**FIGURE 5:**
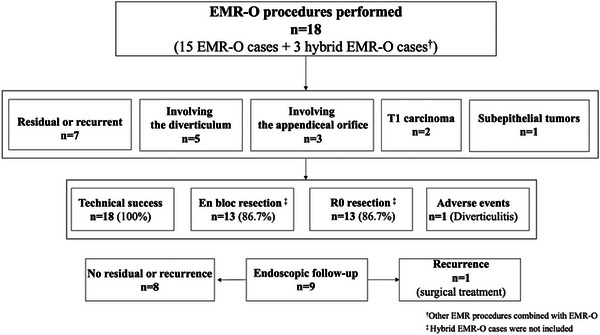
Study flowchart.

Between September 2017 and January 2024, 18 cases were considered suitable for EMR‐O (Figure 5). The median patient age was 72 years (range, 50–81 years). Five (27.8%) patients were administered antithrombotics. The median lesion size was 11 mm (range, 5–25 mm). The lesion was located in the appendiceal orifice in three (16.7%) patients, the colon in 11 (61.1%) patients, and the rectum in four (22.2%) patients. Seven (38.9%) patients had a history of endoscopic treatment. The main indications for EMR‐O were residual or recurrent lesions (38.9%) and diverticular lesions (27.8%). Table 1 shows patient and lesion characteristics as well as indications for EMR‐O.

**TABLE 1:** Patient and lesion characteristics.

Patient (*n* = 18) and lesion characteristics		
Sex, *n* (%)		
Male	14	(77.8)
Female	4	(22.2)
Age, median (range), years,	72	(50–81)
Use of antithrombotic drugs, *n* (%)	5	(27.8)
Lesion size, mm, median (range)	11	(5–25)
Lesion location, *n* (%)		
Appendiceal orifice	3	(16.7)
Cecum	4	(22.2)
Ascending colon	3	(16.7)
Transverse colon	0	(0)
Descending colon	2	(11.1)
Sigmoid colon	2	(11.1)
Rectum	4	(22.2)
Previous treatment, *n* (%)	7	(38.9)
CSP	1	(5.5)
EMR	5	(27.8)
ESD	1	(5.5)
Indication for EMR‐O, *n* (%)		
Residual or recurrent lesion	7	(38.9)
Involving the diverticulum	5	(27.8)
Involving the appendiceal orifice	3	(16.7)
T1 carcinoma	2	(11.1)
Subepithelial tumors	1	(5.5)

Abbreviations: CSP, cold snare polypectomy; EMR, endoscopic mucosal resection; EMR‐O, endoscopic mucosal resection with an over‐the‐scope clip; ESD, endoscopic submucosal dissection.

### Procedure date

The target lesion in all cases was reached using an attached OTSC. The resection method was EMR‐O for 15 (83.3%) cases and hybrid EMR‐O for three (16.7%) cases. The technical success rate was 100%, and the en bloc resection rate was 86.7% (excluding hybrid cases). The median procedure time was 10 min (range, 5–56 min). The median postoperative hospital stay was 2 days (range, 1–9 days). No intraoperative or postoperative complications or postoperative bleeding were observed. One (5.5%) patient developed diverticulitis. No intestinal stenosis was associated with OTSC placement, and no patients required emergency surgery (Table 2).

**TABLE 2:** Procedural data, histopathology, and follow‐up

**Procedural data**		
Target lesion reached with the OTSC, *n* (%)	18	(100)
Procedure		
EMR‐O	15	(83.3)
Hybrid^a^	3	(16.7)
Technical success, *n* (%)	18	(100)
En bloc resection^b^, *n* (%)	13	(86.7)
Procedure time, median (range), min	10	(5–56)
Postoperative hospital stay, median (range), days	2	(2–9)
Adverse events		
Intraoperative and delayed perforation, *n* (%)	0	(0)
Delayed bleeding, *n* (%)	0	(0)
Diverticulitis, *n* (%)	1	(5.5)
Stenosis after OTSC placement, *n* (%)	0	(0)
Need for emergency surgery, *n* (%)	0	(0)
**Histopathology**		
Resected specimen size, median (range), mm	15	(5–27)
Pathological tumor size, median (range), mm	10.5	(4–26)
R0 resection^b^, *n* (%)	13	(86.7)
Pathological resection depth		
Deep submucosal resection, *n* (%)	11	(61.1)
Muscularis resection, *n* (%)	4	(22.2)
Full‐thickness resection, *n* (%)	3	(16.7)
Final histology		
Adenoma	4	(22.2)
Adenocarcinoma	9	(50)
Sessile serrated lesion	2	(11.1)
NET	2	(11.1)
Schwannoma	1	(5.6)
**Follow‐up**		
Follow‐up, median (range), months	3	(1–18)
Endoscopic follow‐up, *n* (%)	9	(50)
Recurrence, *n* (%)	1	(5.5)

Abbreviations: EMR, endoscopic mucosal resection; EMR‐O, endoscopic mucosal resection with an over‐the‐scope clip; NET, neuroendocrine tumor; OTSC, over‐the‐scope clip.

^a^ EMR‐O and other EMR procedures were performed.

^b^ Hybrid cases were not included.

### Histopathology

The median resected specimen size, as assessed by the pathologist, was 15 mm (range, 5–27 mm), and the median tumor size was 10.5 mm (range, 4–26 mm). The R0 resection rate was 86.7% (13/15 cases excluding hybrid cases). Two patients who underwent non‐R0 resection had negative vertical margins. Regarding the resection depth, 11 (61.1%) patients underwent deep submucosal resection, four (22.2%) patients underwent muscle layer resection, and three (16.7%) patients underwent full‐thickness resection. The final histological diagnoses were cancer for nine (50%) cases and adenoma for four (22.2%) cases (Table 2).

### Overview of EMR‐O cases

An overview of all 18 EMR‐O cases is presented in Table 3.

**TABLE 3:** Overview of 15 endoscopic mucosal resection with an over‐the‐scope clip (EMR‐O) cases and three hybrid EMR‐O cases.

Patient no.	Age (years)	Sex	Indication	Location	Lesion size (mm)	Snare size (mm)	Size of OTSC	Cap length of the OTSC (mm)	Procedure time (min)	En block resection	R0 resection	Size of the resected pathological specimen (mm)	Actual tumor size (mm)	Histological diagnosis	Resection depth	Adverse events	Postoperative hospital stay (days)
**EMR‐O**																	
1	70	M	Involving the appendiceal orifice	Appendiceal orifice	8	10	12/6t	6	15	Yes	Yes	13	8	SSL	DR	None	2
2	53	M	Subepithelial tumors	Cecum	6	10	12/6t	6	7	Yes	Yes	10	6	Schwannoma	DR	None	1
3	50	F	Involving the appendiceal orifice	Appendiceal orifice	15	15	14/6t	6	15	Yes	Yes	16	15	SSL	MR	None	1
4	66	M	Residual lesion	Rectum	5	10	12/6t	6	10	Yes	Yes	10	5	NET	DR	None	2
5	79	M	Residual lesion	Rectum	5	15	12/6t	6	10	Yes	Yes	16	5.5	Adenocarcinoma	MR	None	3
6	71	M	Involving the diverticulum	Sigmoid colon	15	10,15	12/6t	6	56	No	No (HMX)	15	15	Adenoma	DR	None	2
7	76	F	Involving the diverticulum	Cecum	15	10	12/6t	6	19	Yes	Yes	15	15	Adenoma	DR	None	2
8	73	M	Involving the diverticulum	Ascending colon	12	15	12/6t	12	15	Yes	Yes	15	13	Adenocarcinoma	FTR	None	2
9	79	M	T1 carcinoma	Descending colon	10	10	12/6t	12	10	Yes	Yes	27	11	Adenocarcinoma	FTR	None	3
10	67	M	Recurrent lesion	Cecum	15	10	12/6t	12	27	No	No (HMX)	20	4.5	Adenocarcinoma	DR	None	2
11	71	M	Residual lesion	Rectum	8	10	12/6t	12	6	Yes	Yes	17	5	Adenocarcinoma	DR	None	2
12	75	M	T1 carcinoma	Descending colon	11	15	14/6t	12	8	Yes	Yes	22	6	Adenocarcinoma	DR	None	2
13	81	M	Residual lesion	Cecum	10	15	12/6t	12	5	Yes	Yes	15	10	Adenoma	FTR	None	2
14	53	F	Residual lesion	Rectum	5	10	11/6t	12	5	Yes	Yes	10	4	NET	MR	None	2
15	67	F	Involving the appendiceal orifice	Appendiceal orifice	14	15	12/6t	12	9	Yes	Yes	16	12	Adenoma	DR	None	2
**Hybrid EMR‐O**																	
16	79	M	Involving the diverticulum	Ascending colon	25	15	12/6t	6	10	No (Hybrid)	No (HMX)	22	22	Adenocarcinoma	DR	None	3
17	80	M	Involving the diverticulum	Sigmoid colon	25	15	12/6t	12	20	No (Hybrid)	Yes	25	14	Adenocarcinoma	MR	None	2
18	81	M	Recurrent lesion	Ascending colon	15	10	12/6t	12	5	No (Hybrid)	No (HMX)	15	5	Adenocarcinoma	DR	Diverticulitis	9

Abbreviations: DR, deep submucosal resection; EMR‐O, endoscopic mucosal resection with an over‐the‐scope clip; F, female; FTR, full‐thickness resection; HMX, horizontal margins were not evaluable; hybrid, underwater EMR and EMR‐O; M, male; MR, muscularis resection; N/A, not assessed; NET, neuroendocrine tumor; OTSC, over‐the‐scope clip; R0, complete resection; SSL, sessile serrated lesion.

### Follow‐up and recurrence

A follow‐up colonoscopy was scheduled based on the final histological diagnosis. Colonoscopy is usually performed within 6 months for cases involving HMX and after 1 year for cases involving R0 resection. At our hospital, endoscopic follow‐up was performed for nine (50%) cases. Follow‐up of the remaining nine cases was performed by a primary care physician. The median follow‐up period was 3 months (range, 2–18 months; Table 2). Recurrence was observed in one patient (patient 18 in Table 3) after hybrid EMR‐O treatment.

## DISCUSSION

In this study, we investigated the treatment outcomes of colorectal EMR‐O performed for 18 patients. EMR‐O has been described by case reports^28^, ^29^; however, to the best of our knowledge, no studies have summarized EMR‐O performed for colorectal tumor cases.

The main indications for EMR‐O are residual, recurrent, and diverticular lesions, and technical success has been achieved for 100% of cases. Generally, EMR/ESD for lesions that are difficult to treat endoscopically can be challenging because long procedure times may be required and complications such as perforation can occur.^16^, ^17^, ^18^, ^30^, ^31^ In contrast, EMR‐O has a shorter procedure time and higher success rate, which we believe are attributable to the simplicity of the procedure and preliminary simulations. In the unlikely event of an OTSC placement error, an OTSC remover (remOVE system; Ovesco Endoscopy) can be used; additionally, OTSC amputation can be considered.^32^

**FIGURE 6:**
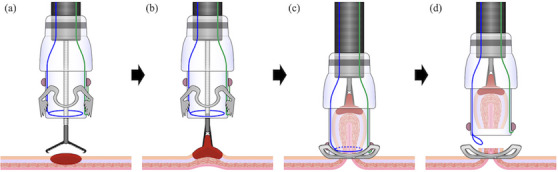
Schema of the full‐thickness resection device (FTRD). After the FTRD is advanced to the lesion, the grasping forceps are advanced through the working channel. The lesion is pulled into the cap of the FTRD using the grasping forceps. The clip is deployed. The lesion above the clip is resected using a snare.

In recent years, EFTR using the FTRD for lesions that are difficult to treat endoscopically has been reported.^19^, ^20^, ^22^, ^23^, ^24^ However, the FTRD has not been approved in Japan. The FTRD cap is longer than the standard OTSC cap (23 mm vs. 6 mm). Therefore, the FTRD can capture lesions up to 30 mm in diameter^19^ (Figure 6). Therefore, its standards and procedure steps are completely different from those of EMR‐O, which targets lesions approximately 15 mm in diameter. Because of the large size of the FTRD, it may not be able to reach some lesion sites and may cause perforation during insertion.^19^, ^33^ Moreover, because the use of the FTRD involves a one‐step procedure that proceeds directly to resection after OTSC placement, perforation may occur when incorrect clip placement or snare malfunction occurs.^19^, ^23^, ^34^ In contrast, EMR‐O reached the lesion in all cases (100% success rate), although the preliminary simulation contributed to its success. We also considered that perforation did not occur because EMR‐O is a two‐step procedure; the lesion is resected only after proper clip placement and snaring of the whole lesion are confirmed.

A previous study of FTRD reported technical success, R0 resection, and adverse events rates of 88.2–89.5%, 76.9–80%, and 9.9–12.2%, respectively.^19^, ^22^, ^23^, ^35^ Although the numbers of cases differed in our study, EMR‐O had a technical success rate of 100% and R0 resection rate of 86.7%, which were comparable to those of procedures involving the FTRD. However, lesions involving the appendiceal orifice pose the risk of appendicitis^36^ and should be limited to post‐appendectomy cases. A few reports of diverticulitis caused by the FTRD exist. The reason for diverticulitis was not mentioned; however, our patient (patient 18 in Table 3) developed diverticulitis 2 days after hybrid EMR‐O. This patient had several diverticula around the target lesion, and we considered that the OTSCs blocked some of the diverticula and caused inflammation attributable to the accumulation of stool in the diverticulum; therefore, antibiotics treatment was administered and the patient recovered well. Awareness of post‐treatment diverticulitis is necessary when diverticula are present around the target lesion. According to previous reports,^19^, ^20^, ^21^, ^22^, ^23^ the residual rate of FTRD was as high as more than 15%. In this study, the residual rate of EMR‐O was 5.5%. In contrast to the FTRD, which is used for a one‐step procedure, EMR‐O involves a two‐step procedure with adjustment of the snare and resection while the lesion margin is evaluated, resulting in a low residual rate.

FTRD is suitable for lesions < 20 mm^19^; however, cases involving the resection of larger lesions using a hybrid procedure comprising EMR and the FTRD have been reported.^37^, ^38^ In this study, there were three cases for which planned piecemeal resection using a hybrid technique comprising UEMR and EMR‐O was performed for lesions that spread inside and outside the diverticulum (patients 16 and 17 in Table 3) and recurrent lesions (patient 18 in Table 3). However, one patient (patient 18) experienced recurrence in the scar area after the hybrid EMR‐O. Initially, the first choice of treatment for patient 18 was surgery because the lesion was recurrent. However, because the patient was elderly, the avoidance of surgery was strongly desired. Therefore, endoscopic treatment was performed. Because the patient experienced recurrence in the scar area 3 months after hybrid EMR‐O, surgery was performed as an additional treatment, and the case was completely cured. Hybrid EMR‐O makes it difficult to evaluate the horizontal margins at the dividing line; therefore, careful follow‐up is necessary.

**FIGURE 7:**
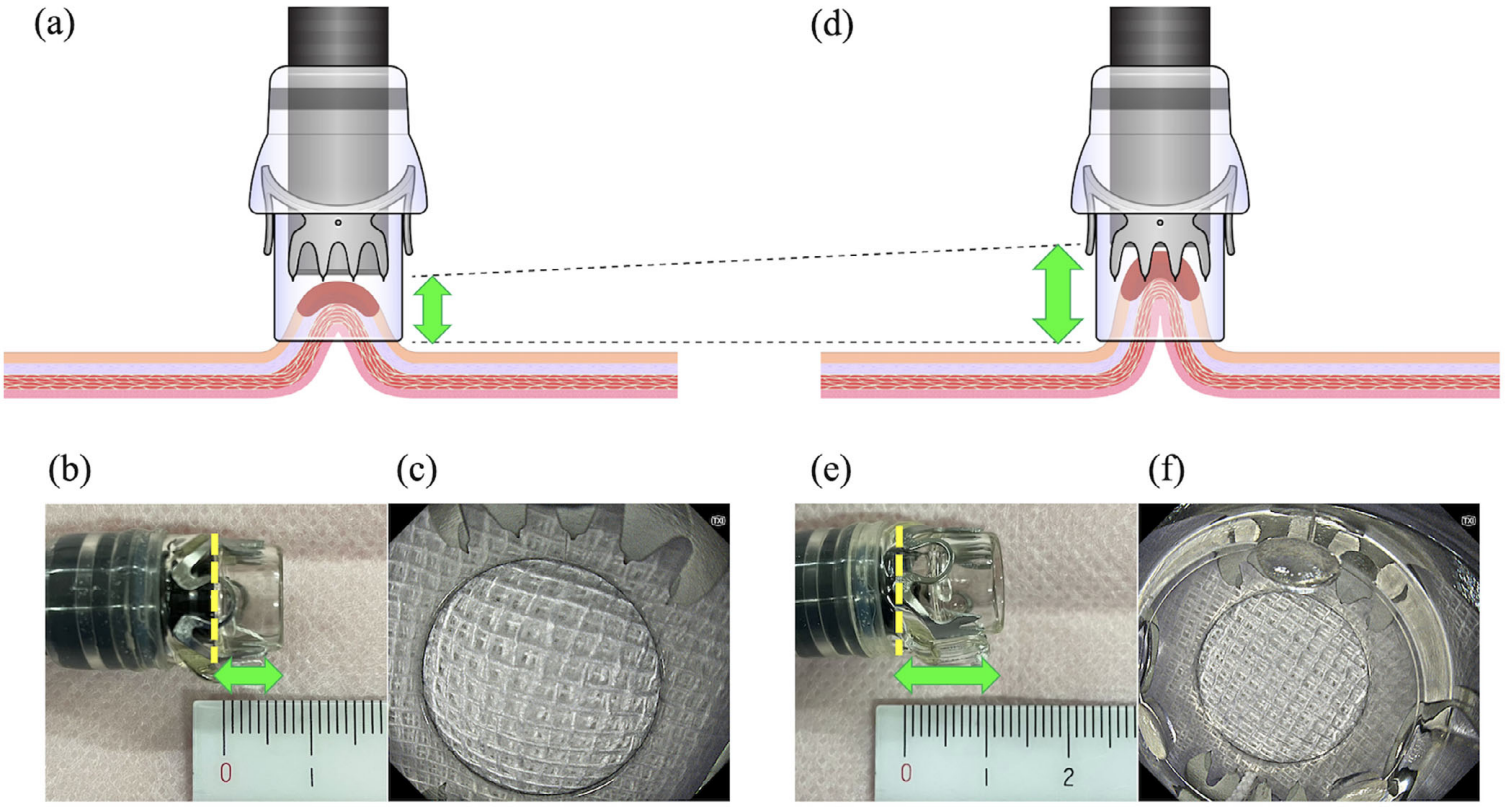
Advanced endoscopic mucosal resection (EMR) with an over‐the‐scope clip (OTSC; EMR‐O). (a) Schema of normal EMR‐O. (b) Appearance of normal EMR‐O. The cap length (from the endoscope tip to the tip of the OTSC cap) is 6 mm. (c) Endoscopic field of view for normal EMR‐O. (d) Schema of advanced EMR‐O. (e) Appearance of advanced EMR‐O. The cap length (from the endoscope tip to the tip of the OTSC cap) is 12 mm. (f) Endoscopic field of view for advanced EMR‐O. The field of view is slightly narrower than that of normal EMR‐O, but it does not interfere with the procedure.

Nevertheless, EMR‐O has several disadvantages. First, the target lesions are limited to small lesions approximately ≤15 mm. Second, the operator cannot adjust the depth of resection. EMR‐O does not reliably guarantee the resection depth below the muscular layer; however, EMR‐O is considered for SETs in the submucosal layer that are observed with endoscopic ultrasonography. In the present study, resection deeper than the muscular layer was performed for seven of the 18 cases (38.7%). When the OTSC is mounted normally, the cap length is set to 6 mm. Nevertheless, we recently improved the technique by doubling the cap length to approximately 12 mm to increase the suction width, thereby increasing the diameter and depth of lesion resection (Figure 7). With this innovation, full‐thickness resection is possible (Table 3). Therefore, the standard setting for the cap length is now 12 mm. NETs in the lower gastrointestinal tract often occur in the rectum; in Japan, ESD and EMR with a ligation device are the established standard treatments for such cases.^39^, ^40^ Technically, NETs can be resected using EMR‐O, but we excluded NETs as an indication for such treatment. Finally, OTSCs are expensive (approximately $800). In Japan, OTSCs are only covered by insurance when used for gastrointestinal perforation, fistulae closure, and hemostasis. Therefore, endoscopic procedures such as EMR‐O are not covered by insurance, and the cost is paid by the hospital. This is a disadvantage; however, we plan to conduct a multicenter prospective study to demonstrate the usefulness of EMR‐O to overcome this financial issue.

This study had some limitations. First, the sample size was limited because this was a single‐center retrospective study; therefore, selection bias may have influenced the results. Second, EMR‐O was not compared with other endoscopic techniques such as EMR and ESD. Furthermore, because this study only revealed short‐term treatment results, data regarding long‐term treatment results are not available. Therefore, we believe that we should increase the sample size at multiple institutions, conduct a prospective study to compare EMR and ESD and analyze the long‐term results.

## CONCLUSION

Although EMR‐O is limited by the target lesion size, it shortens the procedure time, prevents perforation, and avoids the need for surgery. When the FTRD is unavailable, EMR‐O can be a minimally invasive treatment option for small lesions that are difficult to treat using endoscopy. Comparative studies including other endoscopic resection methods are required to further validate the safety and efficacy of EMR‐O.

## CONFLICT OF INTEREST STATEMENT

Takao Itoi is the Editor‐in‐Chief of *Digestive Endoscopy Open*. The other authors declare no conflict of interest.

## ETHICS STATEMENT

This study was approved by the Ethics Review Committee of Saitama Medical University International Medical Center (application number: 19–315).

## PATIENT CONSENT STATEMENT

Owing to the retrospective nature of this study and the fact that written informed consent was not obtained from each patient, a document declaring an opt‐out policy was posted on the Saitama Medical University International Medical Center website, allowing any patient to withdraw from the study.

## CLINICAL TRIAL REGISTRATION

N/A

## Supporting information



Video S1

## Data Availability

The data that support the findings of this study are available from the corresponding author, Tomoaki Tashima, upon reasonable request.
